# Nature Inspired Computing: An Overview and Some Future Directions

**DOI:** 10.1007/s12559-015-9370-8

**Published:** 2015-11-30

**Authors:** Nazmul Siddique, Hojjat Adeli

**Affiliations:** School of Computing and Intelligent Systems, Ulster University, Northland Road, Londonderry, BT48 7JL UK; Departments of Biomedical Engineering, Biomedical Informatics, Civil, Environmental, and Geodetic Engineering, Electrical and Computer Engineering, Neuroscience, and Neurology, The Ohio State University, 470 Hitchcock Hall, 2070 Neil Avenue, Columbus, OH 43210 USA

**Keywords:** Nature-inspired computing, Physics-based algorithms, Biology-based algorithms, Meta-heuristic algorithms, Search and optimisation

## Abstract

This paper presents an overview of significant advances made in the emerging field of nature-inspired computing (NIC) with a focus on the physics- and biology-based approaches and algorithms. A parallel development in the past two decades has been the emergence of the field of computational intelligence (CI) consisting primarily of the three fields of neural networks, evolutionary computing and fuzzy logic. It is observed that NIC and CI intersect. The authors advocate and foresee more cross-fertilisation of the two emerging fields.

## Inspiration from the Nature

Nature does things in an amazing way. Behind the visible phenomena, there are innumerable invisible causes hidden at times. Philosophers and scientists have been observing these phenomena in the nature for centuries and trying to understand, explain, adapt and replicate the artificial systems. There are innumerable agents and forces within the living and non-living world, most of which are unknown and the underlying complexity is beyond human comprehension as a whole. These agents act in parallel and very often against each other giving form and feature to nature, and regulating the harmony, beauty and vigour of life. This is seen as the dialectics of nature which lies in the concept of the evolution of the natural world. The evolution of complexity in nature follows a distinctive order. There is also information processing in nature performed in a distributed, self-organised and optimal manner without any central control [1]. This whole series of forms, mechanical, physical, chemical, biological and social, is distributed according to complexity from lower to higher. This sequence expresses its mutual dependence and relationship in terms of structure and history. The activities change due to changed circumstances. All these phenomena known or partially known so far are emerging as new fields of science and technology, and computing that study problem-solving techniques inspired by nature as well as attempts to understand the underlying principles and mechanisms of natural, physical, chemical and biological organisms that perform complex tasks in a befitting manner with limited resources and capability.

Science is a dialogue between the scientists and the nature [2] which has evolved over the centuries enriching with new concepts, methods and tools and developed into well-defined disciplines of scientific endeavour. Mankind has been trying to understand the nature ever since by developing new tools and techniques. The field of nature-inspired computing (NIC) is interdisciplinary in nature combining computing science with knowledge from different branches of sciences, e.g. physics, chemistry, biology, mathematics and engineering, that allows development of new computational tools such as algorithms, hardware, or wetware for problem-solving, synthesis of patterns, behaviours and organisms [3, 4]. This Keynote paper presents an overview of significant advances made in the emerging field of nature-inspired computing (NIC) with a focus on the physics- and biology-based approaches and algorithms.

## Search and Optimisation

All the living and non-living world, the planetary, galactic, stellar system and the heavenly bodies in the universe belong to nature. One common aspect can be observed in nature, be it physical, chemical or biological, that the nature maintains its equilibrium by any means known or unknown to us. A simplified explanation of the state of equilibrium is the idea of optimum seeking in nature. There is optimum seeking in all spheres of life and nature [5–7]. In all optimum seeking, there are goals or objectives to be achieved and constraints to be satisfied within which the optimum has to be found [8–11]. This optimum seeking can be formulated as an optimisation problem [12–15]. That is, it is reduced to finding the best solution measured by a performance index often known as objective function in many areas of computing and engineering which varies from problem to problem [16–19].

**Fig. 1:**
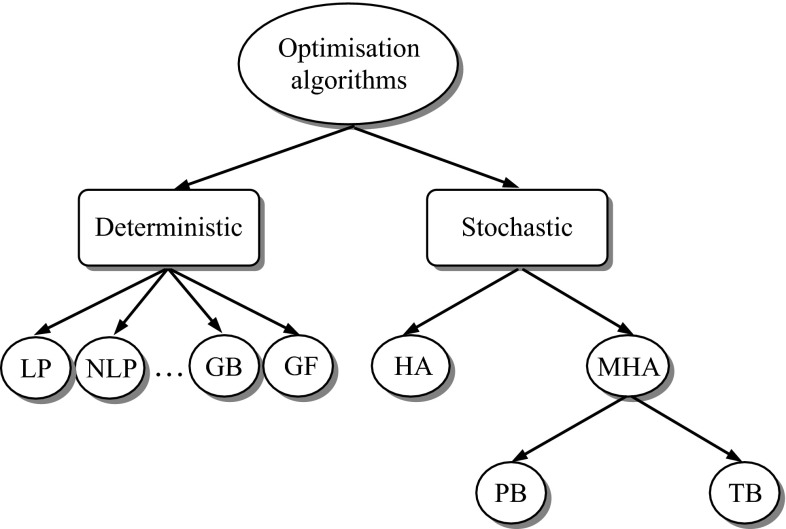
Classification of optimisation algorithms

Many methods have emerged for the solution of optimisation problems which can be divided into two categories based on the produced solutions [20], namely deterministic and nondeterministic (stochastic) algorithms as shown in Fig. 1. Deterministic algorithms in general follow more rigorous procedures repeating the same path every time and providing the same solution in different runs. Most conventional or classic algorithms are deterministic and based on mathematical programming. Many different mathematical programming methods have been developed in the past few decades. Examples of deterministic algorithms are linear programming (LP), convex programming, integer programming, quadratic programming, dynamic programming, nonlinear programming (NLP), and gradient-based (GB) and gradient-free (GF) methods. These methods usually provide accurate solutions for problems in a continuous space. Most of these methods, however, need the gradient information of the objective function and constraints and a suitable initial point.

On the other hand, nondeterministic or stochastic methods exhibit some randomness and produce different solutions in different runs. The advantage is that these methods explore several regions of the search space at the same time and have the ability to escape from local optima and reach the global optimum. Therefore, these methods are more capable of handling NP-hard problems (i.e. problems that have no known solutions in polynomial time) [21]. There are a variety of derivative-free stochastic optimisation algorithms which are of two types: heuristic algorithms (HA) and meta-heuristic algorithms (MHA) (Fig. 1).

Heuristic means to find or discover by means of trial and error. Alan Turning was one of the first to use heuristic algorithms during the Second World War and called his search methods heuristic search. Glover [22] possibly revived the use of heuristic algorithms in 1970s. The general problem with heuristic algorithms (e.g. scatter search) is that there is no guarantee that optimal solutions are reached though quality solutions are found in a reasonable amount of time. The second generation of the optimisation methods is meta-heuristic proposed to solve more complex problems and very often provides better solutions than heuristic algorithms. The 1980s and 1990s saw a proliferation of meta-heuristic algorithms. The recent trends in meta-heuristic algorithms are stochastic algorithms with certain trade-off of random and local search. Every meta-heuristic method consists of a group of search agents that explore the feasible region based on both randomisation and some specified rules. These methods rely extensively on repeated evaluations of the objective function and use heuristic guidelines for estimating the next search direction. The guidelines used are often simple, and the rules are usually inspired by natural phenomena or laws. Glover and Kochenberger [23] present a review of the field of meta-heuristics up to 2003.

There are different classifications of meta-heuristic algorithms reported in the literature [24, 25]. They can be classified as population based (PB) and neighbourhood or trajectory based (TB) (Fig. 1). Neighbourhood-based meta-heuristics such as simulated annealing [26] and tabu search [27] evaluate only one potential solution at a time and the solution moves through a trajectory in the solution space. The steps or moves trace a trajectory in the search space, with nonzero probability that this trajectory can reach the global optimum. In the population-based meta-heuristics, a set of potential solutions move towards goals simultaneously. For example, genetic algorithm (GA) [28, 29] and particle swarm optimisation (PSO) [30, 31] are population-based algorithms and use a population of solutions.

## Nature-Inspired Computing Paradigm

The nature-inspired computing paradigm is fairly vast. Even though science and engineering have evolved over many hundred years with many clever tools and methods available for their solution, there is still a diverse range of problems to be solved, phenomena to be synthesised and questions to be answered. In general, natural computing approaches should be considered when:

- The problem is complex and nonlinear and involves a large number of variables or potential solutions or has multiple objectives.
- The problem to be solved cannot be suitably modelled using conventional approaches such as complex pattern recognition and classification tasks.
- Finding an optimal solution using traditional approaches is not possible, difficult to obtain or cannot be guaranteed, but a quality measure exists that allows comparison of various solutions.
- The problem lends itself to a diversity of solutions or a diversity of solutions is desirable.

**Fig. 2:**
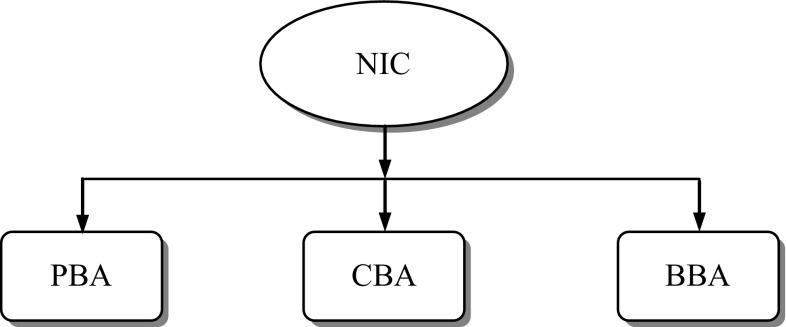
Broad classification of NIC

Nature-inspired computing (NIC) refers to a class of meta-heuristic algorithms that imitate or are inspired by some natural phenomena explained by natural sciences discussed earlier. A common feature shared by all nature-inspired meta-heuristic algorithms is that they combine rules and randomness to imitate some natural phenomena. Many nature-inspired computing paradigms have emerged in recent years. They can be grouped into three broad classes: physics-based algorithms (PBA), chemistry-based algorithms (CBA) [32] and biology-based algorithms (BBA) (Fig. 2).

### Physics-Based Algorithms

Physics-inspired algorithms employ basic principles of physics, for example, Newton’s laws of gravitation, laws of motion and Coulomb’s force law of electrical charge discussed earlier in the paper. They are all based on deterministic physical principles. These algorithms can be categorised broadly as follows:

- Inspired by Newton’s laws of motion, e.g. Colliding Bodies Optimisation (CBO),
- Inspired by Newton’s gravitational force, e.g. Gravitational Search Algorithm (GSA), Central Force Optimisation (CFO), Space Gravitation Optimisation (SGO) and Gravitational Interaction Optimisation (GIO)
- Inspired by celestial mechanics and astronomy, e.g. Big Bang–Big Crunch search (BB–BC), Black Hole Search (BHS), Galaxy-based Search Algorithm (GbSA), Artificial Physics-based Optimisation (APO) and Integrated Radiation Search (IRS),
- Inspired by electromagnetism, e.g. Electromagnetism-like Optimisation (EMO), Charged System Search (CSS) and Hysteretic Optimisation (HO),
- Inspired by optics, e.g. Ray Optimisation (RO),
- Inspired by acoustics, e.g. Harmony Search Algorithm (HSA),
- Inspired by thermodynamics, e.g. Simulated Annealing (SA),
- Inspired by hydrology and hydrodynamics, e.g. Water Drop Algorithm (WDA), River Formation Dynamics Algorithm (RFDA) and Water Cycle Algorithm (WCA).

The earliest of all these algorithms was the Simulated Annealing (SA) algorithm based on the principle of thermo-dynamics [26]. The algorithm simulates the cooling process by gradually lowering the temperature of the system until it converges to a steady state. The idea to use simulated annealing to search for feasible solutions and converge to an optimal solution was very stimulating and led researchers to explore other areas of physics.

An idea from the field of sound and acoustics led to the development of HSA inspired by a phenomenon commonly observed in music. The concept behind the HSA is to find a perfect state of harmony determined by aesthetic estimation [33]. A review of harmony search algorithms and its variants is provided by Siddique and Adeli [34]. Hybrid harmony search algorithms are presented by Siddique and Adeli [35]. Applications of HSA are reviewed in Siddique and Adeli [36].

Zaránd et al. [37] proposed a method of optimisation inspired by demagnetisation, called hysteretic optimisation (HO). This is a process similar to simulated annealing where the material achieves a stable state by slowly decreasing the temperature. That is, finding the ground states of magnetic samples is similar to finding the optimal point in the search process. Based on the principles of electromagnetism, Birbil and Fang [38] introduced the electromagnetism-based optimisation. The EM-based algorithm imitates the attraction–repulsion mechanism of the electromagnetism theory in order to solve unconstrained or bound constrained global optimisation problems. It is called electromagnetism-like optimisation (EMO) algorithm. A solution in EMO algorithm is seen as a charged particle in the search space and its charge relates to the objective function value.

Motivated by natural physical forces, Spears et al. [39] introduced the Artificial Physics Optimisation (APO) where particles are seen as solutions sampled from the feasible region of the problem space. Particles move towards higher fitness regions and cluster to optimal region over time. Heavier mass represents higher fitness value and attracts other masses of lower fitness values. The individual with the best fitness attracts all other individuals with lower fitness values. The individuals with lower fitness values repel each other. That means the individual with best fitness has the biggest mass and move with lower velocity than others. Thus, the attractive–repulsive rule can be treated as the search strategy in the optimisation algorithm which ultimately leads the population to search the better fitness region of the problem. In the initial state, individuals are randomly generated within the feasible region. In APO, mass is defined as the fitness function for the optimisation problem in question. A suitable definition of mass of the individuals is necessary.

Central Force Optimisation (CFO) uses a population of probes that are distributed across a search space [40]. The basic concept of the CFO is the search for the biggest mass that has the strongest force to attract all other masses distributed within a decision space towards it considered as the global optimum of the problem at hand. A review of articles on CFO and its applications to various problems is presented in a recent article by Siddique and Adeli [41].

Gravitational Search Algorithm (GSA) is a population-based search algorithm inspired by the law of gravity and mass interaction [42]. The algorithm considers agents as objects consisting of different masses. The entire agents move due to the gravitational attraction force acting between them, and the progress of the algorithm directs the movements of all agents globally towards the agents with heavier masses [42]. Gravitational Interactions Optimisation (GIO) is inspired by Newton’s law [43]. It has some similarities with GSA and was introduced around the same time independently of GSA. The gravitational constant *G* in GSA decreases linearly with time, whereas GIO uses a hypothetical gravitational constant *G* as constant. GSA uses a set of best individuals to reduce computation time, while GIO allows all masses to interact with each other.

Based on the simple principle of continuous collision between bodies, Kaveh and Mahdavi [44] proposed the Colliding Bodies Optimisation (CBO). Hsiao et al. [45] proposed an optimal searching approach, called Space Gravitational Optimisation (SGO) using the notion of space gravitational curvature inspired by the concept of Einstein equivalence principle. SGO is an embryonic form of CFO [46]. Based on the notion of Big Bang and shrinking phenomenon of Big Crunch, Erol and Eksin [47] proposed Big Bang and Big Crunch (BB–BC) algorithm. In the Big Bang phase, a population of masses is generated with respect to centre of mass. In the Big Crunch phase, all masses collapse into one centre of mass. Thus, the Big Bang phase explores the solution space, while Big Crunch phase performs necessary exploitation as well as convergence. Chuang and Jiang [48] proposed Integrated Radiation Optimisation (IRO) inspired by the gravitational radiation in the curvature of space–time. Hosseini [49] proposed Galaxy-based Search Algorithm (GbSA) inspired by the spiral arm of spiral galaxies to search its surrounding. GbSA uses a spiral-like movement in each dimension of the search space with the help of chaotic steps and constant rotation around the initial solution. The spiral optimisation (SpO) is a multipoint search for continuous optimisation problems. The SpO model is composed of plural logarithmic spiral models and their common centre [50].

Inspired by the phenomenon of the black hole, Hatamlou [51] proposed the Black Hole (BH) algorithm where candidate solutions are considered as stars and the solution is selected to be black hole. At each iteration, the black hole starts attracting other stars around it. If a star gets too close to the black hole, it will be swallowed and a new star (candidate solution) is randomly generated and placed in the search space to start a new search.

The basic idea of Snell’s law is utilised in Ray Optimisation (RO) proposed by Kaveh and Khayatazad [52] where a solution consisting of a vector of variables is simulated by a ray of light passing through space treated as media with different refractive indices. Based on the principles of hydrodynamics and water cycles, Intelligent Water Drop (IWD) was proposed by Shah-Hosseini [53]. Considering the natural phenomenon of river formations through land erosion and sediment deposits, Rabanal et al. [54] proposed River Formation Dynamics (RFD). Eskandar et al. [55] proposed Water Cycle Algorithm (WCA) based on the principle of water cycle that forms streams and rivers where all rivers flow to the sea which is the ultimate destination and optimal solution in terms of optimisation.

### Biology-Based Algorithms

**Fig. 3:**
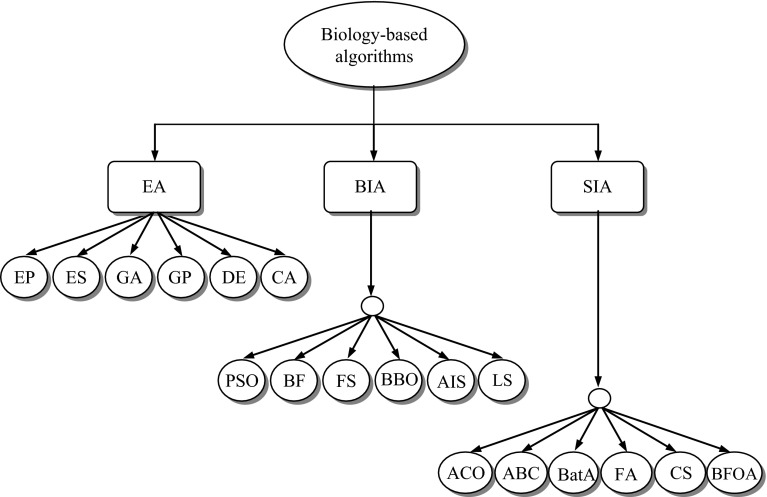
Classification of biology-based algorithms

Biology-based algorithms can be classified into three groups: Evolutionary Algorithms (EA), Bio-inspired Algorithms (BIA) and Swarm Intelligence-based Algorithms (SIA) (Fig. 3).

The fundamental idea of evolutionary algorithms is based on Darwin’s theory of evolution, which gained momentum in the late 1950s nearly a century after publication of the book ‘Origin of Species’. Fraser [56] first conducted a simulation of genetic systems representing organisms by binary strings. Box [57] proposed an evolutionary operation to optimising industrial production. Friedberg [58] proposed an approach to evolve computer programs. The fundamental works of Lowrence Fogel [59] in evolutionary programming, John Holland [60] in genetic algorithms, Ingo Rechenberg [61] and Hans-Paul Schwefel [62] in evolution strategies had great influences on the development of evolutionary algorithms and computation as a general concept for problem-solving and as a powerful tool for optimisation. Since the development years of 1960s, the field evolved into three main branches [63]: evolution strategies [64], evolutionary programming and genetic algorithms. In the 1990s there was another set of development in the evolutionary algorithms such as Koza [65] developed genetic programming, Reynolds [66] developed cultural algorithms and Storn and Price [67] developed differential evolution. Evolutionary algorithms have now found wide spread applications in almost all branches of science and engineering [68–70]. Different variants of EAs such as Evolutionary Programming (EP) [71], Evolution Strategies (ES) [72, 73], Genetic Algorithm (GA) [74–76], Genetic Programming (GP), Differential Evolution (DE) and Cultural Algorithm (CA) are discussed in the book by Siddique and Adeli [77].

The BIA are based on the notion of commonly observed phenomenon in some animal species and movement of organisms. Flocks of birds, herds of quadrupeds and schools of fish are often shown as fascinating examples of self-organised coordination [1, 78]. Particle Swarm Optimisation (PSO) simulates social behaviour of swarms such as birds flocking and fish schooling in nature [79–81]. Particles make use of the best positions encountered and the best position of their neighbours to position themselves towards an optimum solution [82]. There are now as many as about 20 different variants of PSO [83–86].

Bird Flocking (BF) is seen as feature of coherent manoeuvring of a group of individuals due to advantages for protecting and defending from predators, searching for food, and social and mating activities [87]. Natural flocks maintain two balanced behaviours: a desire to stay close to the flock and a desire to avoid collisions within the flock [88]. Reynolds [89] developed a model to mimic the flocking behaviour of birds using three simple rules: collision avoidance with flockmates, velocity matching with nearby flockmates and flock centring to stay close to the flock [90, 91]. Fish School (FS) shows very interesting features in their behaviour. About half the fish species are known to form fish schools at some stage in their lives. FS is observed as self-organised systems consisting of individual autonomous agents [92, 93] and come in many different shapes and sizes [87, 94, 95].

MacArthur and Wilson [96] developed mathematical models of biogeography that describe how species migrate from one island to another, how new species arise and how species become extinct. Since 1960s biogeography has become a major area of research that studies the geographical distribution of biological species. Based on the concept of biogeography, Simon [97] proposed Biogeography Based Optimisation (BBO). Based on the principles of biological immune systems, models of Artificial Immune Systems (AIS) were proposed by Farmer et al. [98] in the 1980s that stipulated the interaction between antibodies mathematically. In 1968, Lindenmayer [99] introduced formalism for simulating the development of multi-cellular organisms, initially known as Lindenmayer systems and subsequently named L-systems which attracted the interest of theoretical computer scientists. Aono and Kunii [100] and Smith [101] used L-systems to create realistic-looking images of trees and plants. There are other bio-inspired search and optimisation algorithms reported in the literature which haven’t attract much attention in the research community such as atmosphere clouds model [102], dolphin echolocation, Japanese tree frogs calling, Egyptian vulture, flower pollination algorithm, great salmon run, invasive weed optimisation, paddy field algorithm, roach infestation algorithm and shuffle frog leaping algorithm.

The SIA are based on the idea of collective behaviours of insects living in colonies such as ants, bees, wasps and termites. Researchers are interested in the new way of achieving a form of collective intelligence called swarm intelligence. SIAs are also advanced as a computational intelligence technique based around the study of collective behaviour in decentralised and self-organised systems. The inspiring source of Ant Colony Optimisation (ACO) is based on the foraging behaviour of real ant colonies [103, 104]. While moving, ants leave a chemical pheromone trail on the ground. When choosing their way, they tend to choose paths marked by strong pheromone concentrations. The pheromone trails will guide other ants to the food source. It has been shown that the indirect communication between the ants via pheromone trails enables them to find the shortest paths between their nest and food sources.

Honey bees search for food sources and collect by foraging in promising flower patches. The simple mechanism of the honey bees inspired researchers to develop a new search algorithm, called Bee Algorithm [105, 106]. Similarly, Artificial Bee Colony (ABC) algorithm was proposed by Karaboga [107] and virtual bee algorithm was proposed by Yang [108]. Bat Algorithm (BatA) is based on the echolocation behaviour of bats. The capability of micro-bats is fascinating as they use a type of sonar, called echolocation, to detect prey, avoid obstacles and locate their roosting crevices in the dark. Yang [109] simulated echolocation behaviour of bats. Quite a number of cuckoo species engage the obligate brood parasitism by laying their eggs in the nests of host birds of different species. Yang and Deb [110] describe the Cuckoo Search (CS) algorithm based on the breeding behaviour of certain cuckoo species. The flashing of fireflies in the summer sky in the tropical regions has been attracting the naturalists and researchers for many years. The rhythm, the rate and the duration of flashing form part of the signalling system that brings two fireflies together. Based on some idealised rules, Yang [111] proposed the Firefly Algorithm (FA).

Individual and groups of bacteria forage for nutrients, e.g. chemotactic (foraging) behaviour of *E. coli* bacteria. Based on this concept, Passino [112] proposed Bacterial Foraging Optimisation Algorithm (BFOA). There are many swarm intelligence-based search and optimisation algorithms reported in the literature which haven’t attract much attention in the research community such as wolf search, cat swarm optimisation, fish swarm optimisation, eagle strategy, krill herd, monkey search and weightless swarm algorithms.

## Conclusion

It is obvious from this review that the field of nature-inspired computing is large and expanding. This invited paper provided a brief summary of significant advances made in this exciting area of research with a focus on the physics- and biology-based approaches and algorithms.

A parallel development has been the emergence of the field of computational intelligence (CI) mainly consisting of neural networks [113–120], evolutionary computing [121] and fuzzy logic [122–125] in the past twenty years starting with the seminal book of Adeli and Hung [126] which demonstrated how a multi-paradigm approach and integration of the three CI computing paradigms can lead to more effective solutions of complicated and intractable pattern recognition and learning problems. It is observed that NIC and CI intersect. Some researchers have argued that swarm intelligence provides computational intelligence. The authors advocate and foresee more cross-fertilisation of the two emerging fields. Evolving neural networks is an example of such cross-fertilisation of two domains [127, 128].
